# Transparent conductive oxides in photoanodes for solar water oxidation

**DOI:** 10.1039/c9na00700h

**Published:** 2020-01-10

**Authors:** Yuanxing Fang, Daniel Commandeur, Wei Cheat Lee, Qiao Chen

**Affiliations:** State Key Laboratory of Photocatalysis on Energy and Environment, College of Chemistry, Fuzhou University Fuzhou 350116 P. R. China yxfang@fzu.edu.cn; Department of Chemistry, School of Life Sciences, University of Sussex Brighton BN1 9RH UK Qiao.Chen@sussex.ac.uk

## Abstract

Rational designs of the conductive layer below photocatalytic films determine the efficiency of a photoanode for solar water oxidation. Generally, transparent conductive oxides (TCOs) are widely used as a conductive layer. In this mini review, the fundamentals of TCOs are explained and typical examples of nanoscale TCOs are presented for application in photoelectrochemical (PEC) water oxidation. In addition, hybrid structures formed by coating other photocatalysts on nanoscale TCOs are discussed. In the future, the nanostructured electrode may inspire the design of a series of optoelectronic applications.

## Abstract 2

Rational designs of the conductive layer below photocatalytic films determine the efficiency of a photoanode for solar water oxidation.

## Graphical abstract

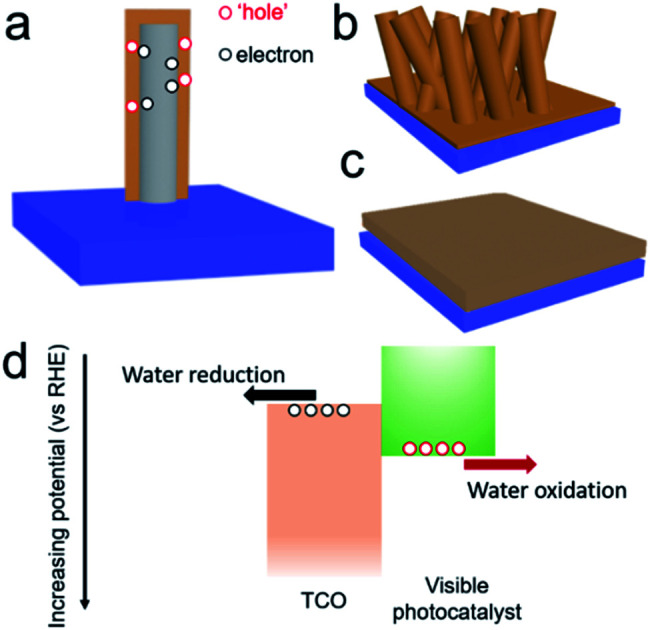

## Introduction

Water splitting using PEC systems has received increasing interest, because this conversion fulfils the sustainable goal for the storage of solar energy in chemical bonds.^1–4^ The approach avoids the carbon cycle, and it therefore leads to almost zero impact on the environment.^5,6^ Water oxidation at photoanode is normally the rate determining reaction for overall water splitting, since this oxidation reaction involves 4 electrons and 2 oxygen atoms.^7^ To achieve an efficient photoanode, three general issues should be considered in the order (1) light absorption, (2) charge separation/transport and (3) surface reactions.^8,9^ Among them, charge separation and transport are of particular importance. As the oxidation and reduction reactions are physically separated in two different chambers,^10^ the photoexcited electron from the photoanode must migrate through the semiconducting films to the cathode for hydrogen production.^11^ In other words, low conductivity of the semiconducting films could extensively limit the performance due to the effect of charge recombination. For instance, a pristine hematite photoanode presented excellent visible light absorption up to 590 nm, but the minority carrier diffusion length is short (2–4 nm) and thus hinders its efficiency.^12^ Therefore, its solar conversion efficiency is far below the state-of-the-art.^13^ What is worse, the incident photoenergy would release in other forms of non-collective energies, including thermal energy and photoluminescence energy. The emissions of these energies not only reduce the PEC conversion efficiency, but also degrade the photoactive films themselves, and thus likely reduce the working life.^14^ As such, a few strategies were developed to overcome this issue, for instance, the improvement of the crystallinity and optimization of conductivity with doping.^15–17^

Beyond the photocatalytic films, the conductive substrate is the other key part of the PEC electrode, and the materials of which are normally TCOs.^18–21^ For a typical photoanode, TCO films normally bind the photocatalytic films and the substrate support to collect photoexcited electrons, which are then transferred to the cathode on the other side for the reductive reaction.^22,23^ Typical TCOs include fluorine/indium doped tin oxide (FTO/ITO) and aluminum doped zinc oxide (AZO), which are already commercialized by coating flat films on glass or polymer substrates.^24^ In the past few years, novel designs of TCO textures were also developed to promote charge separation and transfer and internal light scattering.

**Fig. 1:**
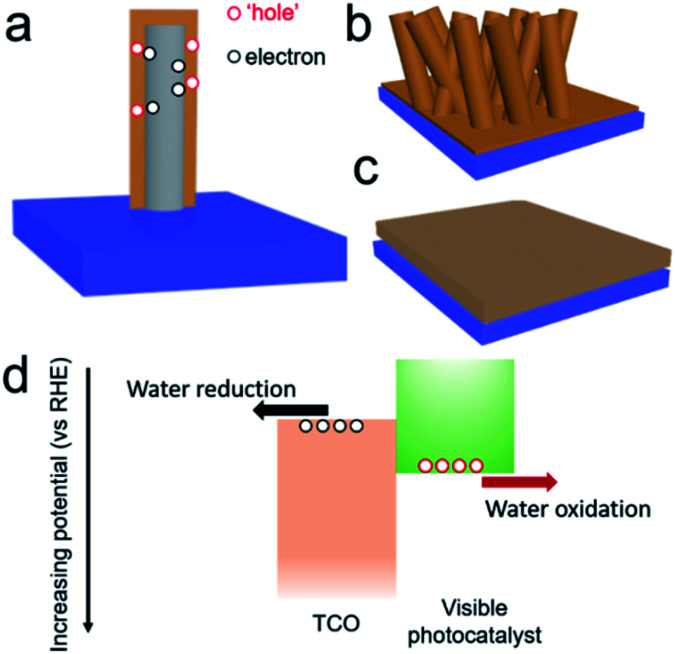
(a) Illustration of the hybrid visible photoanode by coating a visible light photocatalyst on TCOs. Comparison of (b) 1D hybrid structure with (c) flat films. (d) Illustration of a type II hetero-structured photoanode by coating a visible photocatalyst on TCOs for solar water oxidation.

An example of one-dimensional (1D) TCO nanorods (NRs) and the corresponding hybrid structure are shown in Fig. 1b. In a comparison of flat films (Fig. 1c), this nanostructure, on one hand, could increase the effective interfacial surface area for improving the rate of charge injection and increases the area for surface reactions (Fig. 1a). On the other hand, a careful selection of the material for the formation of the hybrid structure would encourage charge migration to the cathode and minimize the recombination probability. Type II hetero-band structures of the photoanode are ideal for solar water oxidation (Fig. 1d), where the photoexcited electrons are transferred to the TCOs and carried to the cathode with a sufficient reduction potential. The appropriate relative positions of the electronic states are significant to achieve an efficient photoanode for solar water oxidation.^25^ Despite a few reports being presented focusing on the TCOs in photoanodes, a comprehensive analysis is still absent. The merit of TCOs for photoanodes should be analyzed; only then can the possible way be proposed for further developing them in photoelectrodes and other optoelectronic applications.

In this mini review, the designs of nanoscale TCOs are reviewed for the development of efficient photoanodes for solar water oxidation. The principle of TCOs is introduced. Typical examples of nanostructured TCOs and their synthesis are presented. In addition, the hybrid structures formed by coating visible-active photocatalytic films on nanostructured TCOs are also reviewed, highlighting the favorable band structures for improving charge separation and transfer, thus optimizing the performance of solar water oxidation. Along this line, the importance of 1D TCOs in PEC systems is emphasized. In the future, the prototypical nanostructured electrodes may inspire a series of optoelectronic applications.^26–28^

### The fundamentals of TCO materials

Excellent TCO materials should only absorb light shorter than 400 nm, so that they are visible light transparent. The ideal free carrier concentration should be above 10^19^ cm^−3^.^29^ The use of TCOs for electronics was initially realized for a transparent display, which can be traced back to the 1930s, when H.G. Wells imagined such a material in his fiction novel called ‘The Shape of Things to Come’.^29^ With modern technology, this prototypical material is widely applied in electronics, such as mobile phones, electronic skin, solar cells and many more. Many such metal oxides have large bandgaps which only absorb in the ultra-violet (UV) spectrum. The materials are mainly based on SnO,^55^ TiO_2_,^56,57^ In_2_O_3_,^58,59^ ZnO^60,61^ and more, which have bandgap values of 3.49, 3.20, 3.00 and 3.20 eV, respectively. However, the pristine semiconducting metal oxides normally present limited diffusion length for the minority charges with insufficient conductivity.^62,63^

**Fig. 2:**
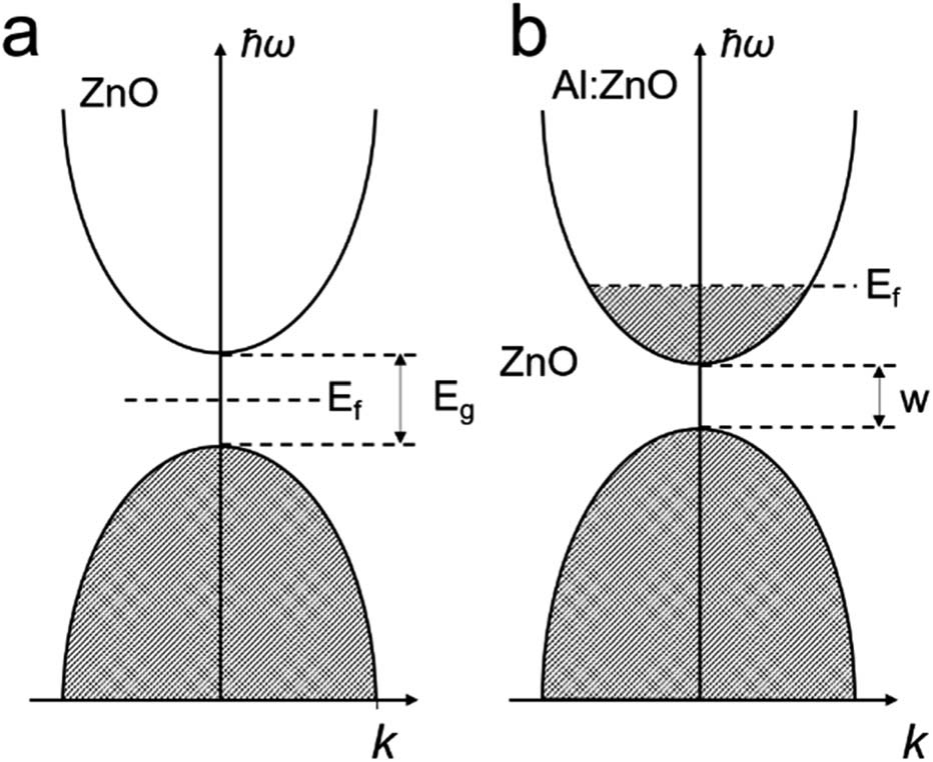
The illustration of band structures of (a) ZnO and (b) Al:ZnO.

In principle, improving their conductivity is realized by introducing a shallow donor/withdrawer.^29,64^ Such doping would not lead to significant differences in the band edges, but injected electrons can be readily transported by the shallow donor or withdrawer. Whether to use a shallow donor or withdrawer depends on the type of semiconductor (p- or n-types) and the properties of the dopants. For example, in AZO,^37^ the major composition of ZnO is an n-type semiconductor. When doping Al^3+^ into ZnO, Al ions replace Zn ions in the ZnO crystal to introduce extra electrons as shallow donors in the conduction band near the band edge, as shown in Fig. 2, which allows the charge to migrate (Fig. 2b). Despite the wide range of TCO materials available, limited species of TCOs were used for PEC applications. Most of the metal oxide based TCO materials and the corresponding dopants are summarized in Table 1 and their nanostructures can be developed for highly effective PEC water splitting in the near future.

**Table 1:** Typical metal oxides and dopants for TCOs^29^

Metal oxides	Dopants
SnO_2_	Sb,^30^ F,^31^ As,^32^ Nb,^33^ Ta^33^
TiO_2_	Nb,^34^ Ta,^35^ In^36^
ZnO	Al,^37^ B,^38^ Cl,^39^ Y,^40^ V,^41^ Si,^42^ Ti,^43^ Zr^44^
CdO	In,^45^ Sn^46^
In_2_O_3_	Sn,^47^ Mo,^48^ F,^49^ Ti,^50^ Zr,^51^ Nb,^52^ Ta,^53^ W^54^

### From planar to nanoscale TCOs for PEC water oxidation

To the best of our knowledge, Nb doped TiO_2_ (Nb:TiO_2_) films are the first case for the investigation of TCO materials in the PEC water oxidation, reported in 1991.^65^ In this research, the effects of firing temperature, membrane thickness and Nb-doping level on quantum efficiency were examined. The performance of PEC water oxidation is improved with Nb doping, achieving the optimal PEC performance at a doping concentration of *ca.* 5 mol%, when the efficiency for charge separation and transfer approaches was balanced. The principle for the improved charge mobility in Nb:TiO_2_ is similar to that of AZO. TiO_2_ is an n-type semiconductor. Nb was the substitutional dopant in the TiO_2_ crystal and delocalizes charge from Nb onto neighboring Ti ions. In more detail, the Nb 4d orbital would affect the Ti 3d orbital to induce shallow donors, thus resulting in the improved conductivity.

Recently, owing to the rapid developments of nanostructured materials, TCO based nanostructures, especially their 1D nanostructured version, were also widely reported. Nb:TiO_2_ nanotubes were synthesized through traditional self-organizing anodization of Ti–Nb alloys. It is found that with respect to undoped TiO_2_, *ca.* 5 times photocurrent density can be achieved using optimal Nb:TiO_2_ nanotubes (*ca.* 5 at%), resulting in the optimal value of *ca.* 1.0 mA cm^−2^ at the applied voltage bias of 0.5 V *vs.* Ag/AgCl.^66^ Liu and co-workers reported the synthesis of Nb:TiO_2_ NRs through the hydrothermal approach for PEC water oxidation.^67^ They found that by increasing the doping of Nb in the TiO_2_ crystal, the vertical growth of the NR structure would be affected since the Nb ion would modify the crystal surface free energy. With the optimal concentration (0.25% Nb), the highest photocurrent density of *ca.* 0.9 mA cm^−2^ is achieved with a voltage bias of 1.23 eV *vs.* RHE.

**Fig. 3:**
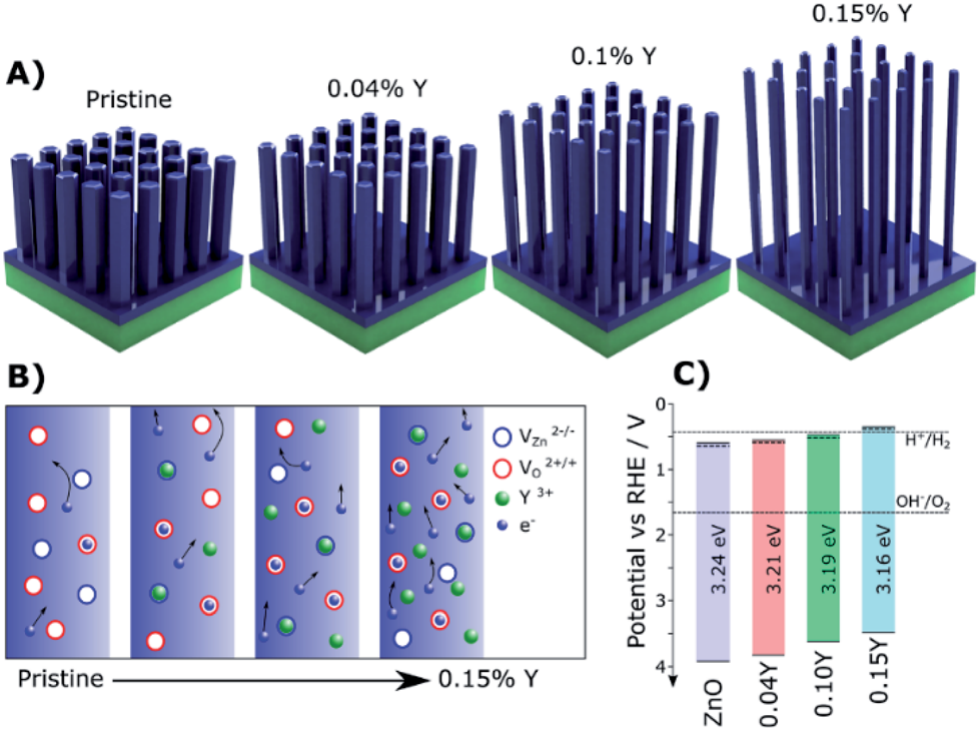
(A) Illustrations of the Y:ZnO NRs with different concentrations of Y. (B) Schematic explaining the increased electron mobility with increasing Y doping concentration. (C) The details of the band structure of pristine ZnO and Y doped ZnO with different concentrations of Y. Reproduced with permission from ref. 68; Copyright (2019) American Chemical Society.

Doped ZnO NRs were also widely investigated for PEC water oxidation due to their conventional synthesis, especially for 1D NRs.^69^ By doping Cl ions, the conductivity of ZnO nanowires could approach that of typical metals identified by current *vs.* voltage (*I*/*V*) measurements.^39^ For PEC water oxidation, when conductive Cl:ZnO was coated on TiO_2_ NRs, they exhibited a significantly enhanced photocurrent density of 2.0 mA cm^−2^ at 0 V *vs.* the saturated calomel electrode (SCE). Yttrium doped ZnO (Y:ZnO) NRs were synthesized using the hydrothermal approach. The conductivity of the Y:ZnO NRs were investigated with a four-point probe, resulting in a conductivity of *ca.* 0.84 Ω cm.^40^ More recently, a rapid microwave approach was used to synthesize Y:ZnO NRs and applied for PEC water oxidation.^68^ Upon increasing the doped concentration of Y, the morphology of the NRs becomes thinner and longer (Fig. 3A). This morphology is favourable for solar water oxidation due to the increasing surface area. The mechanism for the improved charge migration is illustrated in Fig. 3B. In addition, the band structure of the pristine ZnO and Y:ZnO samples is also shown in Fig. 3C, revealing that their band edges and gap are mainly preserved after doping. The product presented an optimal photocurrent of 0.84 mA cm^−2^ at 1.23 V *vs.* RHE with 0.1% Y doping. This result corresponds to a 47% enhancement compared to pristine ZnO in solar conversion.

### Hybrid visible light photoanode with nanoscale TCOs

Despite the TCO material approaching high quantum efficiency in photocatalytic water oxidation, the nature of its large bandgaps (>3.00 eV) restricts its solar light absorption within the UV spectrum, corresponding to *ca.* 5% of solar energy.^70^ Therefore, even with much improved charge mobility, this conversion is still far below the requirements (10%) for practical applications. Although increasing the doping level could potentially narrow the bandgap, it is normally accompanied by the reduction of crystallinity. Too many crystal defects could reduce charge mobility and thus decrease photo-oxidation performance. To overcome the wide bandgap issue, a hybrid photoanode is designed by coating narrow bandgap photocatalysts on the surfaces of nanostructured TCOs. The structure offers the benefits of both good electron conductivity and visible light absorption. The nanomorphology will add extra benefit of a large effective surface area to facilitate oxidation at the electrolyte/photocatalyst interfaces. Most of the narrow bandgap photocatalysts have a short charge diffusion length. The design of the hybrid photoanode can effectively avoid such a problem. Within such a hybrid structure, the light absorption is determined by the thickness of the TCO films, while the charge transfer is determined by the thickness of the films of the narrow bandgap photocatalysts. Hence, the light absorption can be maximized without affecting the charge mobility.

**Fig. 4:**
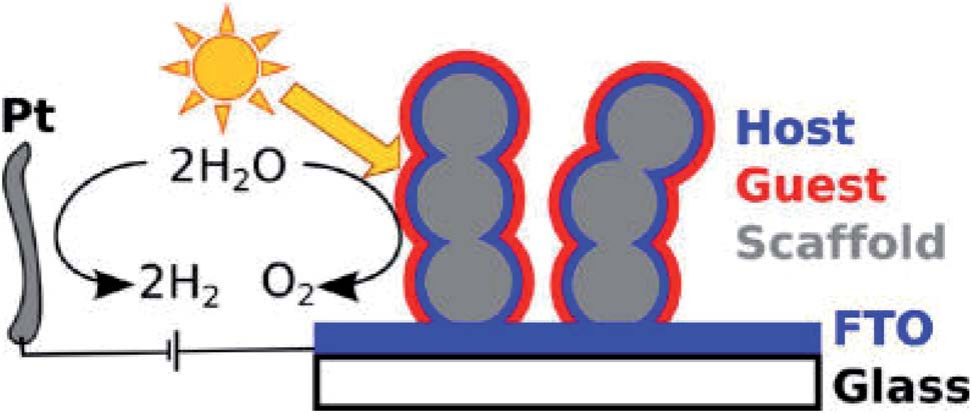
Illustration of the host–guest PEC system by coating hematite films on Nb:SnO_2_. Reproduced with permission from ref. 71; Copyright (2012) American Chemical Society.

A typical example is visible light sensitive hematite, because it has an extremely short diffusion length for charge carriers (*ca.* 2 nm).^13^ As early as 2012, Gratzel and coworkers synthesized a 3D porous Nb:SnO_2_ host electrode to facilitate charge transport and improve the PEC water oxidation efficiency of hematite.^71^ The structure of the photoanode is shown in Fig. 4.^71^ The Nb:SnO_2_ host is fabricated by atomic layer deposition (ALD) and it was crystallized by high temperature annealing to achieve high transparency and conductance with good chemical stability over a wide range of pH. The optimized Nb:SnO_2_ films showed a high electrical conductivity of up to 37 S cm^−1^ concomitant with a low optical attenuation coefficient of 0.99 μm^−1^ at 550 nm. This 3D nano-electrode is used as a host to support the deposited hematite layers on the surface and achieves a photocurrent density of *ca.* 1 mA cm^−2^ with a voltage bias of 1.2 eV (*vs.* RHE). Zou and co-workers reported the synthesis of another core–shell structure by coating hematite on ITO NRs for PEC water oxidation.^72^ ITO NRs were synthesized through chemical vapor deposition (CVD) on a quartz substrate. The hematite layer is then coated on the surface of the ITO NRs, with a layer thickness of 30 to 40 nm. The boundary between hematite and ITO was clearly distinguished with high-resolution transmission electron microscopy (HR-TEM). The hybrid core–shell photoanode reached a current density of *ca.* 1.1 mA cm^−2^ at 1.23 V (*vs.* RHE), which is double that of planar hematite films. The stability of the photoanode was tested in 1 M NaOH aqueous electrolyte under AM1.5 illumination. The photocurrent density was maintained for as long as 40000 s. This result indicated that with improved charge transport, the stability of the photoanode is also significantly improved.

**Fig. 5:**
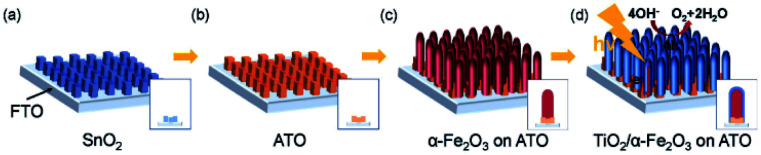
Schematic illustration of the synthesis of the hybrid photoanode. (a) Growth of SnO_2_ NRs, (b) doping Sb into SnO_2_, (c) growth of the hematite NRs and (d) coating TiO_2_ on the as-prepared NRs. Reproduced with permission from ref. 73 Copyright (2018) Wiley-VCH.

Conductive Sb:SnO_2_ NRs were also synthesized by thermal vapor deposition and hematite NRs were grown on the surface of the conductive NRs for enhanced PEC water oxidation. By annealing the hybrid photoanode at 650 °C, a photocurrent density of 0.88 mA cm^−2^ was achieved at 1.23 V (*vs.* RHE). This result is 3 times higher than that of hematite NRs on FTO glass annealed at the same temperature. More recently, an extra TiO_2_ coating was applied on the hematite/Sb:SnO_2_ NRs, as shown in Fig. 5.^73^ This photoanode presented an optimal photocurrent density of *ca.* 1.75 mA cm^−2^, which is double that of the hematite/Sb:SnO_2_ photoanode.

In addition to hematite, TiO_2_ was also widely used as a photocatalyst, due to its negative conduction band minimum. TiO_2_ NRs coated on Sb:SnO_2_ NRs achieved enhanced PEC water oxidation.^74^ In this case, the TiO_2_ NRs were formed on the Sb:SnO_2_ NRs by the chemical bath deposition method. A maximum photocurrent of *ca.* 0.6 mA cm^−2^ was achieved by this hybrid structure. A similar result was achieved by depositing a TiO_2_ layer *via* ALD on the Sb:SnO_2_ nanoparticles on FTO glass as a photoanode, which presented an optimal photocurrent density of *ca.* 0.58 mA cm^−2^ at 1 V (*vs.* RHE) under AM 1.5G illumination.^75^ A similar idea was also reported by replacing Sb:SnO_2_ nanoparticles with FTO colloid films. A further increase of the photocurrent density is achieved (0.7 mA cm^−2^) under the same conditions. The improved PEC efficiency can be attributed to the improved charge collection by the nano FTO colloid films, together with an increased surface area from the nanotextured photoanode.^76^

**Fig. 6:**
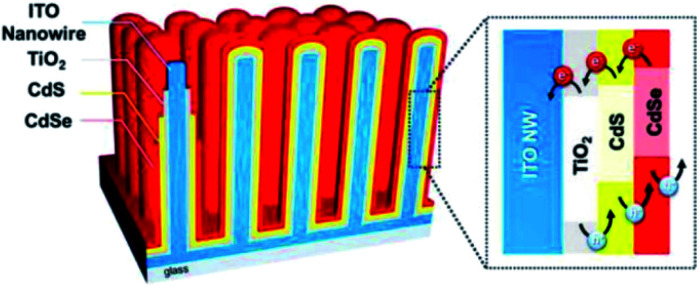
Schematic illustration of the CdSe/CdS/TiO_2_/ITO multi-shell photoanode. Reproduced with permission from ref. 77; Copyright (2016) American Chemical Society.

CdS and CdSe are well established visible-light sensitive photocatalysts and thus have potential for improved solar PEC applications. By integrating them with highly conductive NRs, both good visible light absorption and excellent charge transportation can be achieved. Lee and co-workers^77^ demonstrated the coating of CdS and CdSe based photocatalysts on the outside of vertically aligned conductive ITO NRs for PEC water oxidation. The structure of their photoanode is shown in Fig. 6.^77^ ITO NRs were produced using the CVD approach. A thin layer of TiO_2_ was coated on ITO NRs in order to control the charge recombination on the ITO surface. The photoactive layer of CdS and CdSe was coated by successive ionic layer adsorption and reaction (SILAR) and chemical bath deposition (CBD) methods, respectively. This multi-shell photoanode presented a significant increase in solar conversion with a photocurrent density of 16.2 mA cm^−2^. This result is unexpectedly higher than most of the other similar reports. In this system, three key points determine this high efficiency: (1) the ITO NRs play a key role in charge transport to improve the quantum efficiency; (2) the heterostructure of the bandgap ensures the migration of the electrons and ‘holes’ to the right direction; (3) strong visible light absorption of CdS and CdSe allows the use of the majority of solar illumination.

Recently, Zou and co-workers developed conductive, vertically aligned AZO NRs by doping Al into ZnO NRs through a hydrothermal approach. A CdS layer is then coated on the surface of the AZO NRs by SILAR. Thin Al_2_O_3_ films were further deposited on the surface of the as-prepared core–shell NRs using the magnetron sputtering technique for improving the stability of the NRs. It results in a photocurrent density of *ca.* 10.4 mA cm^−2^ at 1.23 V (*vs.* RHE). Meanwhile, the photocurrent density of an optimal photoanode can be preserved at *ca.* 75% for a 3600 s test, which is excellent for a CdS based photoanode.

BiVO_4_ is a relatively new emerging visible light photocatalyst; the major issue to apply it for PEC applications is also the short diffusion length of minority carriers. The typical approach is to dope Mo into BiVO_4_ to increase charge mobility, which could significantly improve the photocurrent density to *ca.* 2.73 mA cm^−2^. Yang and co-worker doped TiO_2_ NRs with Ta to achieve conductive NRs.^78^ In this research, a solid state diffusion approach based on ALD was used to achieve Ta:TiO_2_. With respect to the traditional hydrothermal method, this method requires a high processing temperature (823 K) to achieve homogeneously doped Ta on the surface of TiO_2_. The quantity of doping can be facilely controlled. The improved charge mobility was measured through electrochemical impedance spectroscopy and analyzed with Mott–Schottky plots. These NRs were further coated with BiVO_4_ nanoparticles to achieve visible light absorption. The valence band edges of Ta:TiO_2_ and BiVO_4_ were identified using ultraviolet photoelectron spectroscopy (UPS). It is shown that a typical type II heterostructure was formed at the interfaces. As such, under light illumination, the photoexcited electrons were ready to migrate from BiVO_4_ to Ta:TiO_2_, which left the ‘holes’ for water oxidation. A photocurrent density of 2.0 mA cm^−2^ was achieved. Here, the hybrid photoanode system has helped to overcome the limitation of the charge diffusion length of the photocatalyst with good chemical stability in comparison with CdS or CdSe photocatalysts.

**Fig. 7:**
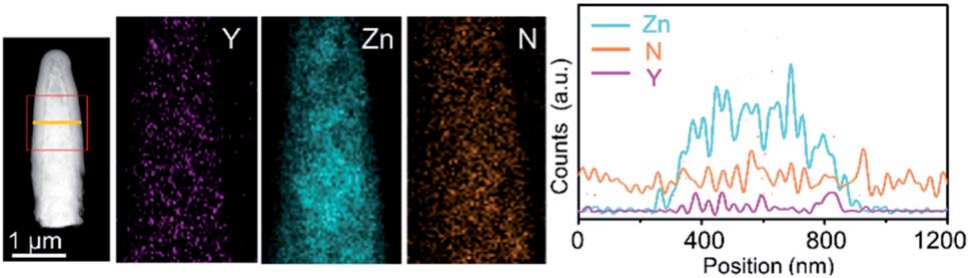
TEM images of PCN coated Y:ZnO NRs and the corresponding distribution of the elements along the rods. Reproduced with permission from ref. 79 Copyright (2018) Wiley-VCH.

Polymeric carbon nitride (PCN) has attracted attention for photocatalysis in the past few years.^80–83^ This metal-free material is also tested as a photoanode for solar water oxidation by forming PCN films on flat TCO films. However, due to the poor charge mobility of the films and insufficient contact between the film and TCO films, the photocurrent density is hindered. PCN presented an improved efficiency for PEC water oxidation,^84^ when it was integrated with highly conductive NRs. Wang and co-workers synthesized Y:ZnO NRs using a hydrothermal approach.^79^ The conductivity of the NRs was determined with a four-point probe. PCN films are then coated on the Y:ZnO NRs by thermal vapor deposition. The transmission electron microscopy image of a single core–shell NR is shown in Fig. 7. The boundary between Y:ZnO and PCN can be clearly observed to identify the composition of the materials. The band structure of both materials (Y:ZnO and PCN) was carefully investigated to illustrate their type II alignment, which would promote charge separation. As a result, they achieved a photocurrent density *ca.* 0.4 mA cm^−2^, which is much better than the reported values for PCN photoanodes (*ca.* 0.1 mA cm^−2^).^85,86^ However, the PEC performance of PCN can be further improved due to the optimizable properties of optoelectronics.

## Conclusions

In conclusion, the design of TCOs applied as photoanodes was reviewed. The excellent conductivity and transparency of TCOs were explained, and typical examples of nanostructured TCOs are presented. In addition, the hybrid structure formed by coating active photocatalysts on nanoscale TCOs was also discussed, highlighting its favorable band structures for visible light absorption while relying on TCOs for improved charge separation and transfer, thus improving the performance of solar water oxidation. The development of nanoscale TCOs might inspire a series of applications in optoelectronics,^87^ sensors,^88^ photocatalysts,^89^ materials sciences^90^ and more.^91–93^

## Conflicts of interest

There are no conflicts to declare.

## Supplementary Material
